# Analysis of the safety and immunogenicity profile of an azoximer bromide polymer-adjuvanted subunit influenza vaccine.

**DOI:** 10.12688/f1000research.75869.2

**Published:** 2022-11-03

**Authors:** Ronald Kompier, Pieter Neels, Walter Beyer, Tim Hardman, Dmitry Lioznov, Susanna Kharit, Michail Kostinov

**Affiliations:** 1 Ruijgenhoeck 6, 2201 EW Noordwijk, Vaccine Consultancy, The Netherlands, Netherlands Antilles; 2 Vaccine Advice BVBA, Zoersel, Belgium; 3 Niche Science and Technology Ltd., Unit 26, Falstaff House, Bardolph Road, Niche Science and Technology, London, UK; 4 Smorodintsev Research Institute of Influenza, Saint Petersburg, Russian Federation; 5 First Pavlov State Medical University, Saint Petersburg, Russian Federation; 6 Scientific Research Institute of Children’s Infections of the Russian Federal Biomedical Agency, St. Petersburg, Russian Federation; 7 Department of Allergology, I.I. Mechnikov Research Institute of Vaccines and Sera, Moscow, Russian Federation; 8 Moscow State Medical University, Department of Epidemiology and Modern Vaccination Technologies, Sechenov First, Moscow, Russian Federation

**Keywords:** influenza vaccine, vaccine adjuvant, azoximer bromide, vaccine safety, immunogenicity, review, meta-analysis

## Abstract

A systematic review of clinical trials conducted with a low-dose inactivated influenza vaccine adjuvanted by azoximer bromide (AZB, Polyoxidonium), was performed to compare vaccine reactogenicity against non-adjuvant vaccines. We also assessed whether lower amounts of antigen per viral strain in AZB-adjuvanted vaccines affected antibody responses. A robust search strategy identified scientific publications reporting 30 clinical trials, comprising data on 11,736 participants and 86 trial arms, for inclusion in the analysis. Local reaction rates (R _lr_) appeared to be lower in AZB-adjuvanted vaccine treatment arms versus comparator vaccine treatment arms. Post-vaccination geometric mean titres in those exposed to AZB-adjuvanted vaccine and comparator vaccine treatment arms were similar in both children and adults aged 18–60 years, implying an antigen-sparing effect by AZB. Meta‑regression analysis based on a literature search of records or reports of clinical trials featuring AZB and the inactivated subunit of influenza published between 1998–2018 was conducted online in January 2019 and updated in August 2019. This search covered trials performed between 1993 and 2016 and suggested that AZB did not contribute to vaccine reactogenicity.

## Introduction

Influenza virus infections cause seasonal epidemics worldwide and continue to be a major health and economic burden. ^1^^–^^3^ Despite ongoing research, understanding of the precise pathogenesis of disease and appropriate specific treatments remain elusive, leaving vaccination as the most effective means of prevention. Current licenced influenza vaccines involve either inactivated, live-attenuated or recombinant formulations and contain either the whole virion, split virion or viral subunits as the antigen. In particular, inactivated subunit (SU) vaccines possess a favourable tolerability and safety profile and are relatively simple to produce. Influenza vaccines aim to induce antibodies against viral hemagglutinin (HA) proteins which undergo steady antigenic drift and therefore require regular vaccine reformulation. ^4^^–^^8^ Furthermore, HA antigens insufficiently induce long-term immunity and may require large doses and/or more than one vaccination to guarantee robust protection. ^9^^–^^12^ Adjuvants can be added to inactivated vaccines to increase their immunogenicity ^13^; this is particularly relevant when considering vaccines for the elderly and immunocompromised people, as well as during pandemics, when a rapid antibody response is required. ^14^ Well-established adjuvants include alum ^15^ and MF59 ^10^^,^^11^; however, efficacy on antibody induction is impacted by age and may vary considerably ^9^^,^^16^^,^^17^

An adjuvanted, inactivated subunit influenza vaccine for subcutaneous and intramuscular injection (Grippol, NPO Petrovax Pharm, Moscow, Russia) has been used in the Russian Federation and other countries of the Commonwealth of Independent States for over two decades. The vaccine contains influenza virus HA and neuraminidase subunits ^18^ adjuvanted by 500 μg azoximer bromide (AZB, Polyoxidonium), an immune-modulating polymer belonging to a class of synthetic heterochain polyamines. Azoximer bromide itself, has a long history of use throughout the Russian Federation and neighbouring countries, both within clinical research and as an additional treatment for a variety of infections. This is due to its beneficial effects on a host’s innate immune responses, including enhancement of phagocytosis; and its anti-toxic effects, such as the reduction of free radicals. ^20^ The exact mechanism(s) by which AZB achieves these effects are not fully understood, with an effect on *melanoma differentiation-associated protein 5 (MDA5)* gene expression via effects on *toll-like receptor 4 (TLR 4)* gene, in addition to direct binding to cellular components of the blood. ^20^ There are three formulations of Grippol, or azoximer-adjuvanted subunit vaccine (AZB-SU): Two trivalent formulations contain HAs of two strains of influenza A (from A/H3N2 and A/H1N1 subtypes) and one of two B strains (from B/Victoria or B/Yamagata lineage). The third formulation contains HAs of all four strains (quadrivalent). All formulations of AZB-SU contain 5 μg HA per strain, a third less than the standard amount of 15 μg HA. ^20^

The addition of AZB to influenza SU vaccine enhances antibody production, even in immunosuppressed mice. ^19^^,^^21^^,^^22^ In the Grippol formulations, AZB allows a reduction in the amount of HA antigen required per dose. ^23^ This antigen-sparing strategy raises two questions:

1. Does AZB itself increase vaccine reactogenicity compared with non-adjuvant vaccines, *i.e.*, does it have an impact on vaccine safety and tolerability?
2. Does AZB-SU induce antibody levels comparable to non-adjuvant vaccines, despite its lower HA dose?

The present study investigated these questions by interrogating the clinical data reported for clinical trials with AZB-SU (published and unpublished) over the past 20 years.

## Methods

### Data identification, selection criteria and extraction

This study was conducted according to the principles of the Preferred Reporting Items for Systematic Reviews and Meta-Analyses (PRISMA). ^24^ A literature search was performed in English- and Russian-language databases (United States National Library of Medicine at the National Institutes of Health PubMed database, Cochrane Database of Systematic Reviews, Russian National Medical Library) to identify any public records or reports of clinical trials conducted with AZB-SU between 1998 and 2018. Relevant articles were identified by searching the terms: ‘influenza’ AND ‘vaccin*’ AND ‘[‘azoximer bromide’ OR ‘polyoxidonium’ OR ‘Grippol’]. The online search was performed in January 2019 and updated in August 2019.

Manual searching of existing lists of references, provided by the manufacturer, NPO Petrovax Pharm (Moscow, Russia), was performed, and assessments included original trial reports and trial compilations produced by the manufacturer. Study reports were rejected if a full text was not openly available or in case of pre-clinical studies, field studies or post-marketing reports on rare events or individual cases.

Reports from studies with at least one of the following assessments were included:

- Reactogenicity: Participants were followed for at least 5–6 days after intervention (vaccine or placebo), and local and systemic vaccine reactions were recorded.
- Safety: Serious adverse events (SAEs) were recorded within at least 3–4 weeks after intervention.
- Immunogenicity: Antibody titres against vaccine strains were determined with a standard hemagglutination inhibition assay, in sera drawn before and 3–4 weeks after intervention.

A data extraction form was used based on the PRISMA recommendations. Titles and abstracts identified from searches were screened by two independent reviewers. They also independently reviewed full-text versions of marked articles that met the predefined criteria. Each study was provided with a unique identifier number (Study reference number). All extracted data were independently reviewed by two researchers and finalised after consultation and agreement on inclusion and exclusion assignment was unanimous. Data extracted from included studies comprised: authors and date of study, population characteristics (including age, medical history and previous vaccinations), trial design and intervention arms, vaccines used (formulations, virus strains, amount of HA per strain and per dose), numbers or percentages of participants with post-vaccination local or systemic reactions and the numbers of participants who experienced SAEs. Immunogenicity data, *i.e.* pre- and post-vaccination geometric mean titre (GMT) and variance ( *e.g.*, confidence interval [CI]) and serologic Committee for Proprietary Medicinal Products (CPMP) variables (see below), were collected for efficacy analyses. All entry data were critically assessed for eligibility by the reviewers.

### Definitions and outcomes

Reactogenicity outcomes per treatment arm were the rate (proportion) of participants with any local (R _lr_) or systemic (R _sr_) reactions up to 6 days post-vaccination. Safety outcomes per treatment arm were the number of SAEs within 4 weeks following vaccination. Only data on SAE widely recognised to be related to vaccination were extracted, such as allergic reactions, Guillain-Barré syndrome and narcolepsy. The primary immunogenicity outcome was pre- and post-vaccination GMT. Post-vaccination HA antibody titre correlates well with protection against influenza infection, and could act as a predictor of actual vaccine efficacy in the wider population using an evidence-based clinical protection curve. ^25^ Secondary efficacy outcomes were: seroprotection rate (SPR, proportion of participants with a post-vaccination titre of at least 40); seroconversion rate (SCR, proportion of participants with at least a 4-fold increase from baseline); mean fold increase (MFI) (post-vaccination GMT: pre-vaccination GMT ratio). For annual re-licensing of inactivated influenza vaccines in the European Union, age-defined criteria for SPR, SCR and MFI in groups of at least 50 vaccinees, had been set by the CPMP in 1992, ^19^ but were withdrawn in 2016. These variables are regarded here for the purpose of completeness.

### Statistical analyses

In trials with randomized allocation of different treatments, the following measures of distance between two treatments, and their 95% CIs, were calculated:

- The local and systemic rate difference (RDlr and RDsr), derived from local and systemic reaction rates, respectively. Two treatments were regarded as having similar reactogenicity when the 95% CI of their RD value included zero.
- The GMT ratio (GMTR), derived from post-vaccination GMTs. Two treatments were regarded as having similar immunogenicity when the 95% CI of their GMTR value includes 1.0. In trials designed and powered to assess non-inferiority or superiority, one treatment was regarded non-inferior to another one when the lower limit of the 95% CI of their GMTR value exceeded 0.67, and superior when it exceeded 1.5.

All participants assessed for efficacy had three or four anti-HA titre values (one titre for each vaccine strain; A-H3N2 and A-H1N1, and one or two B strains), therefore trial arms were subdivided into sub-arms, one sub-arm per vaccine strain. Any sub-arms that were further subdivided into groups with low and high pre-vaccinations titres in original publications were pooled to increase statistical power. When two comparator vaccines were given within a trial, they were pooled into one treatment arm due to their similarity in terms of safety and immunogenicity. ^26^ An additional analysis was performed for one trial with AZB-SU and comparator vaccine whereby GMT and standard deviation (SD) values were transformed into post-vaccination antibody-predicted protection rates (post-PR _ab_). ^27^^,^^28^ The post-PR _ab_ ratio between treatment arms was calculated. The post-PR _ab_ values were regarded similar if the 95% CI of their ratio included 1.0.

A *post-hoc* linear meta-regression analysis was performed to adjust local and systemic reaction rates for several variables: total HA amount per vaccine dose, mean age and health status. Adjusted reaction rates were tested with a dummy binary variable representing AZB content (0: placebo and comparator vaccines [no AZB]; 1: AZB-SU) to determine whether there was any intrinsic reactogenicity associated with AZB. Funnel plots were constructed from logarithmic local and systemic rate ratios and their standard errors to assess potential publication bias, ^29^ which would be by asymmetry in the funnel plot.

Outcome variables from several trials were combined using the inverse-variance weighted method or were subjected to least-squares linear meta-regression using the software package ‘Comprehensive Meta-Analysis’, ^30^ [version 3.3.070/20140 (Biostat, Englewood, NJ, USA)]. Other analyses were performed using IBM SPSS Statistics for Windows version 25/2017 (Armonk, NY, USA).

## Results

### Study selection

**Figure 1.**
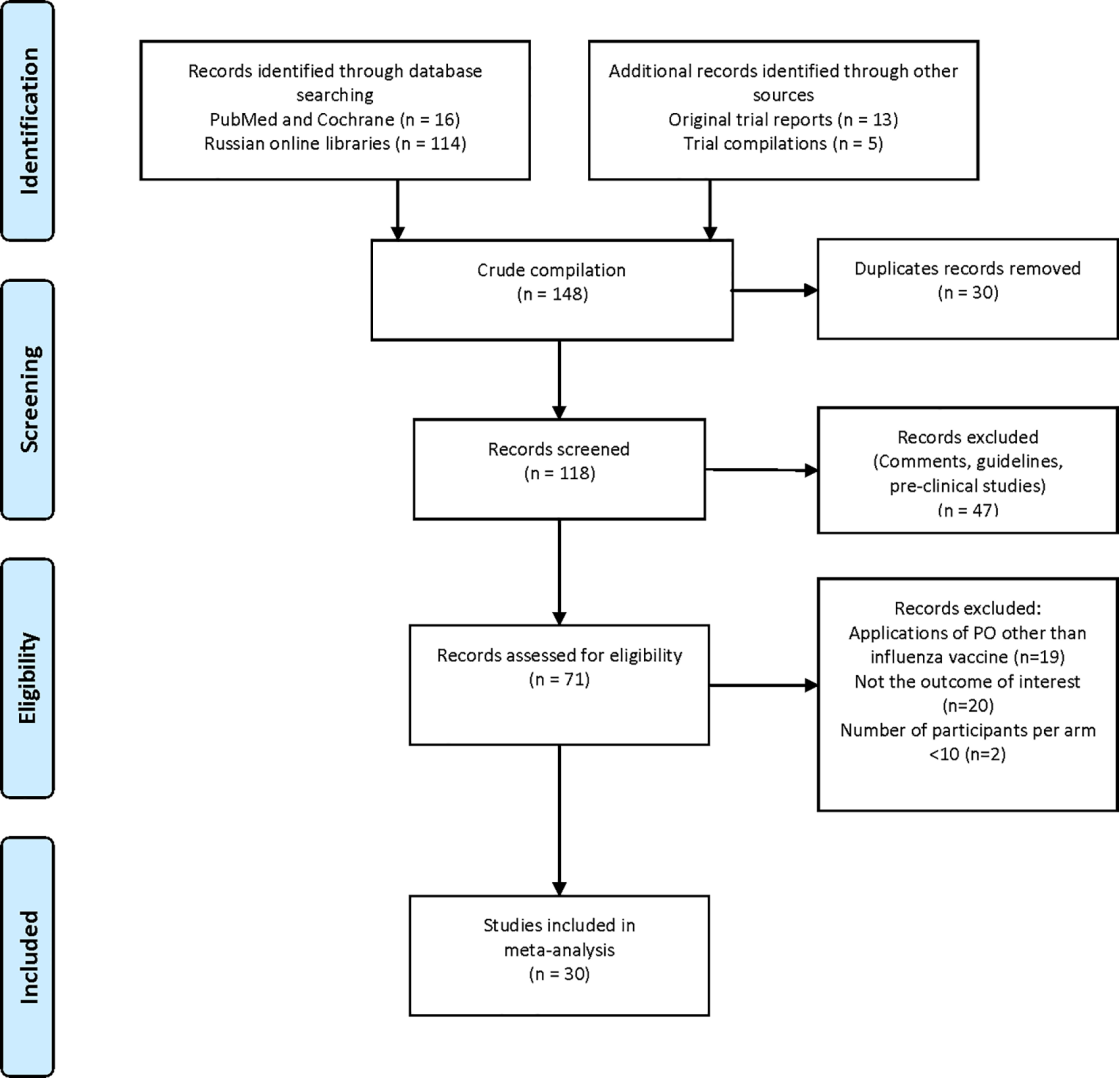
PRISMA flow diagram of literature retrieval. Legend: (none).

The selection process of clinical trials is summarised in Table 1. One hundred and forty-eight reports were identified, of which 30 were found to be duplicates, and 118 records were screened. Forty-seven reports were found not to include clinical trial data and were therefore excluded. Seventy-one records were assessed further for eligibility. Nineteen records were excluded because they did not focus on the topic of interest, 20 records were excluded because they did not include data on the outcome of interest, and two records were excluded because the study arm population was considered to be too small (<10 participants). Data from the remaining 30 publications or reports were included in the analyses ( Figure 1).

**Table 1.** Population characteristics of 30 clinical trials included in the analysis.

All trials	N	Participants N (%)	
	30	11,736	100.0
**Age class (years)**			
Toddlers (0.5 to 3 years)	3	441	3.8
Children/adolescents (3 to 17 years)	12	5140	43.8
Adults (18 – 60 years)	10	3369	28.7
Predominantly elderly (>60 years)	5	2786	23.7
**Health status**			
Predominantly healthy	18	7392	63.0
Predominantly with chronic disease	12	4344	37.0
**Study design**			
Uncontrolled or placebo-controlled stand-alone trial	11	7172	61.1
Randomised bridging trial between AZ-SU formulations	12	2708	23.1
Randomised non-inferiority trial with non-adjuvanted vaccines	7	1856	15.8
**Assessment**			
Both safety and immunogenicity	14	3950	33.7
Safety only	15	7746	66.0
Immunogenicity only	1	40	0.3
**Intervention**			
***AZB-SU formulation***			
AZB-SU _1996_ (with thimerosal)	23	3328	28.4
AZB-SU _2008_ (thimerosal-free)	28	3183	27.1
AZB-SU _TC_ (tissue culture-grown)	3	290	2.5
AZB-SU _2018_ (with both B strains)	2	236	2.0
***Non-adjuvanted comparator vaccine***			
Whole virus IIV	1	108	0.9
Split IIV	9	905	7.7
Subunit IIV	4	378	3.2
***No vaccine***			
Intramuscular placebo (saline)	11	2242	19.1
No relevant intervention	5	1066	9.1

IIV = inactivated influenza vaccine; AZB-SU = polymer-adjuvanted subunit (vaccine).

### Study characteristics

The trials were performed between 1993 and 2016 and comprised 11,736 participants aged between 6 months and 99 years ( Table 1). The majority of participants (7392 [63.0%]) were reported as healthy (no reported chronic disease). The remaining 4344 participants (37.0%) were reported as having allergies, chronic obstructive pulmonary disease, cardiovascular disease, diabetes mellitus type 1, HIV infection or age-related chronic conditions. A breakdown of study population characteristics by trial is presented in *Extended data*, Figure C. Eleven of the trials were uncontrolled or placebo-controlled standalone trials, 12 were randomised bridging trials (between AZB-SU formulations), and seven were randomised non-inferiority trials conducted with non-adjuvanted comparator vaccines. Fourteen trials (46.7%) assessed both safety and immunogenicity, 15 (50.0%) assessed safety only, and one (3.3%) assessed immunogenicity only. A total of 7037 participants (60.0%) in 56 treatment arms received the AZB-SU vaccine and 1391 (11.9%) participants in 15 treatment arms received comparator vaccines, which were either whole virus (Immunopreparation ^®^), split (Begrivac ^®^, Vaxigrip ^®^, Fluarix ^®^), or subunit formulation (Influvac, Agrippal). Participants in trivalent AZB-SU arms received 15 μg HA per dose in most cases, except in dose-finding trials where some participants received lower (7.5 μg HA) or higher (30 μg HA) doses. The HA amount of the influenza B component was increased from 5 μg to 11 μg (21 μg HA per dose) in two trials (T14 and T16, Supplemental Materials Figure D). Participants in quadrivalent AZB-SU arms all received 5 μg HA per vaccine strain (20 μg HA per dose). All comparator vaccines contained 15 μg HA per vaccine strain; participants in comparator treatment arms received 45 μg HA per dose as all comparator vaccines were trivalent. A total of 2242 participants (19.1%) in 10 trial arms received a placebo (saline) and 1066 participants (9.1%) received no intervention. No data were reported for two no-intervention treatment arms, which were therefore excluded from the analysis. Other placebo or no-intervention arms that contained eligible data were included in the safety analysis and excluded from the immunogenicity analysis. Further details on study design and trial arms in each trial are presented in the Supplementary Materials, Figure D ( *Extended data*).

### Safety analysis

**Figure 2.**
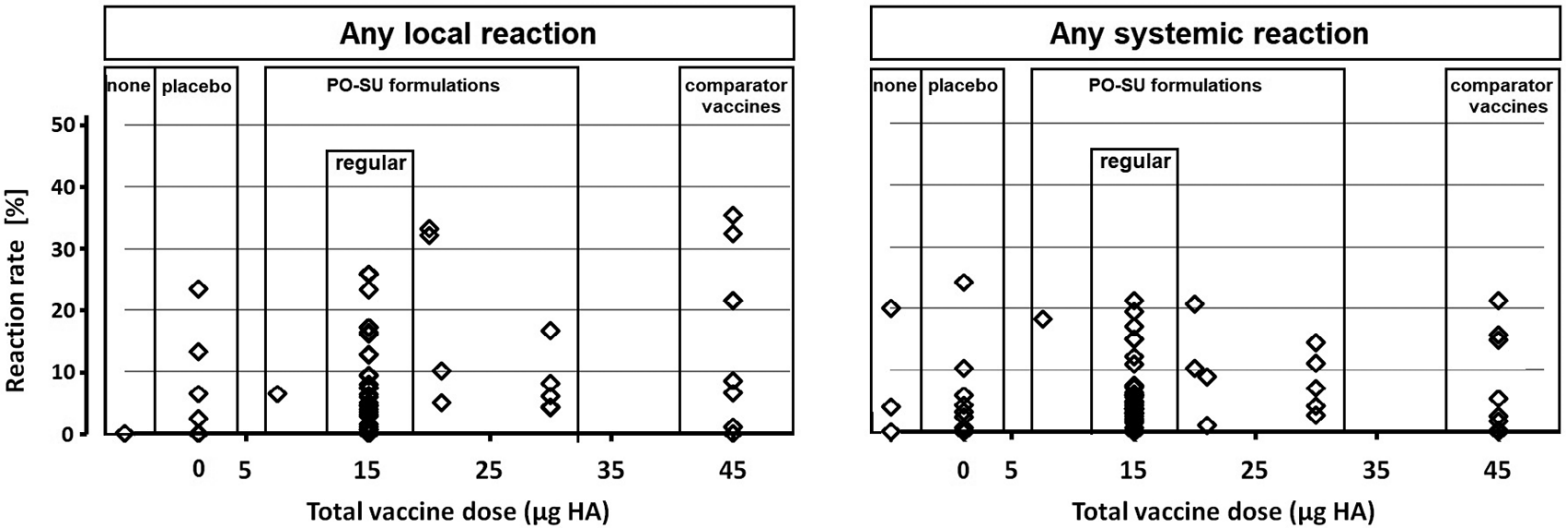
Local and systemic reaction rate estimates from 69 single intervention arms. Legend: Symbols represent local and systemic reaction rate estimates from single intervention arms, arranged according to increasing total vaccine dose.

No SAEs or deaths were reported in any of the trials. Overall, local reactions (at least one) occurred in 646 of 10,405 participants (6.2%), and at least one systemic reaction occurred in 495 of 10,348 participants (4.8%). The difference in number of total participants was a consequence of exclusion of what trial authors identified as intercurrent trivial events that were reported in some of the original papers. Single R _lr_ and R _sr_ values were grouped according to treatment type, and their distributions were plotted against total HA per vaccine dose ( Figure 2) reaching from no HA (placebo and no-intervention arms) to 45 μg HA (comparator vaccines). Reaction rate values (R _lr_ and R _sr_) were <6.0% for most treatment arms. Notably, the largest R _lr_ value (35.5%) occurred in a comparator vaccine treatment arm, and the largest R _sr_ value (24.3%) in a placebo arm.

**Figure 3.**
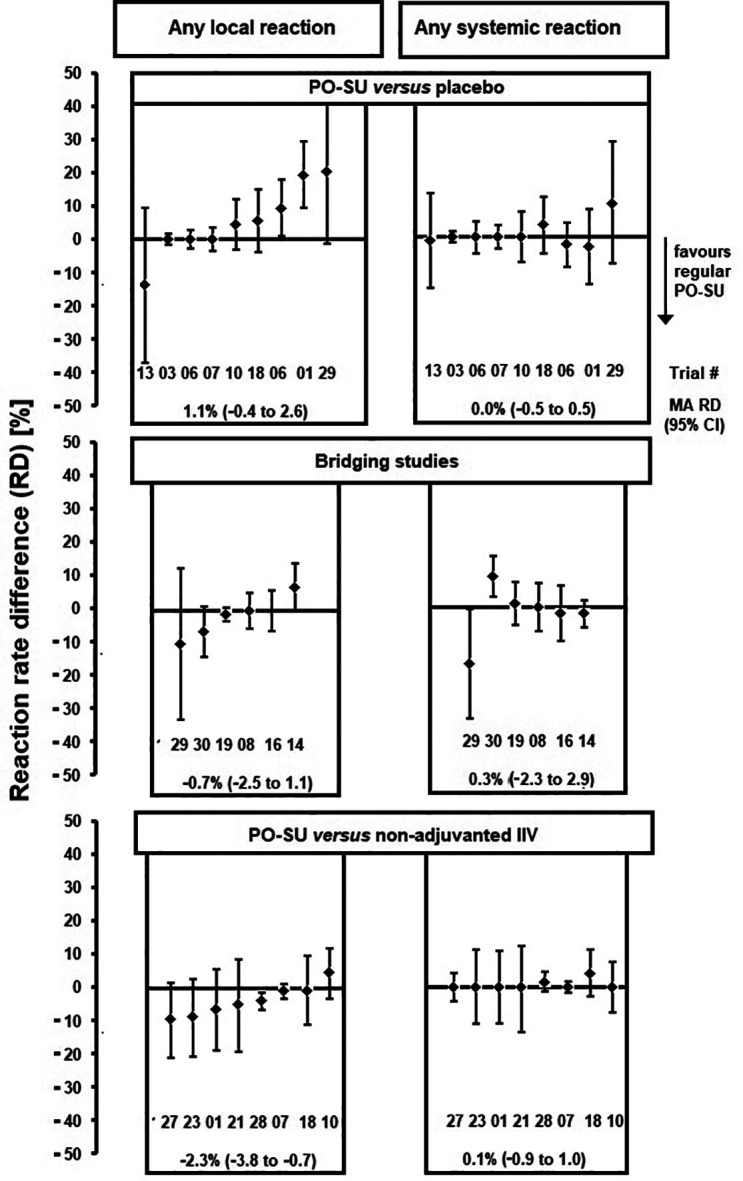
Randomised comparison trials (regular AZB-SU versus three comparator classes). Reaction rate difference values. Legend: Minuend: AZB-SU _1996_ in trials T01, T03, T06, T07, T08, T10, T14 and T16; AZB-SU _2008_ in T13, T18, T19, T21, T23, T27 and T28; AZB-SU _2018_ (20 μg HA) in T29 and T30. Subtrahend bridging studies: AZB-SU _1996_ 2 times 2.5 μg HA in T08; AZB-SU _2008_ in T14, T29 and T30; PO- SU _TC_ in T16 and T19. Subtrahend non-adjuvanted IIV: whole virus in T01; subunit in T10, T18, T21, T23, T27 and T28; subunit and split combined in T07. For trial numbers, see Supplementary Materials, Figure B and C. T06 was divided into two age groups. MA RD, descriptive IV-weighted averages.

In randomised trials, rate differences (RD) between reaction rates of AZB-SU vaccines and other treatment types (placebo or comparator vaccines) could be calculated ( Figure 3). For local reactions, most 95% CIs included 0. However, AZB-SU tended to have lower R _lr_ compared with comparator vaccines and higher R _lr_ compared with placebo. Descriptive IV-weighted average RD _lr_ values differed significantly between AZB-SU and comparator vaccines (-2.3%; 95% CI: -3.8 to -0.7). For systemic reactions, no such trend was observed, and IV-weighted RD _sr_ values were not significantly different between treatment types. Meta-regression analysis of records or reports of clinical trials conducted 1993–2016 and published 1998–2018, identified during an online literature search performed in January 2019 (updated August 2019), suggested a positive correlation between reaction rate and total amount (μg) HA per vaccine dose for local reactions (P=0.03), but not for systemic reactions. There was a positive correlation between both R _lr_ and R _sr_ with mean age up to a mean of 60 years; reaction rates dropped sharply at higher mean ages. There was no correlation between AZB-SU R _lr_ or R _sr_ and health status. Other possible modulators of reactogenicity were not analysed as they were reported in only a few trials. None of the various meta-regression models involving a dummy variable representing AZB content showed evidence of reactogenicity associated with AZB: Reaction rates were similar when adjusted for total HA per dose and mean age ( *Extended data*, Figure E). Funnel plots constructed from logarithmic local and systemic rate ratios were largely symmetrical.

### Immunogenicity

**Figure 4.**
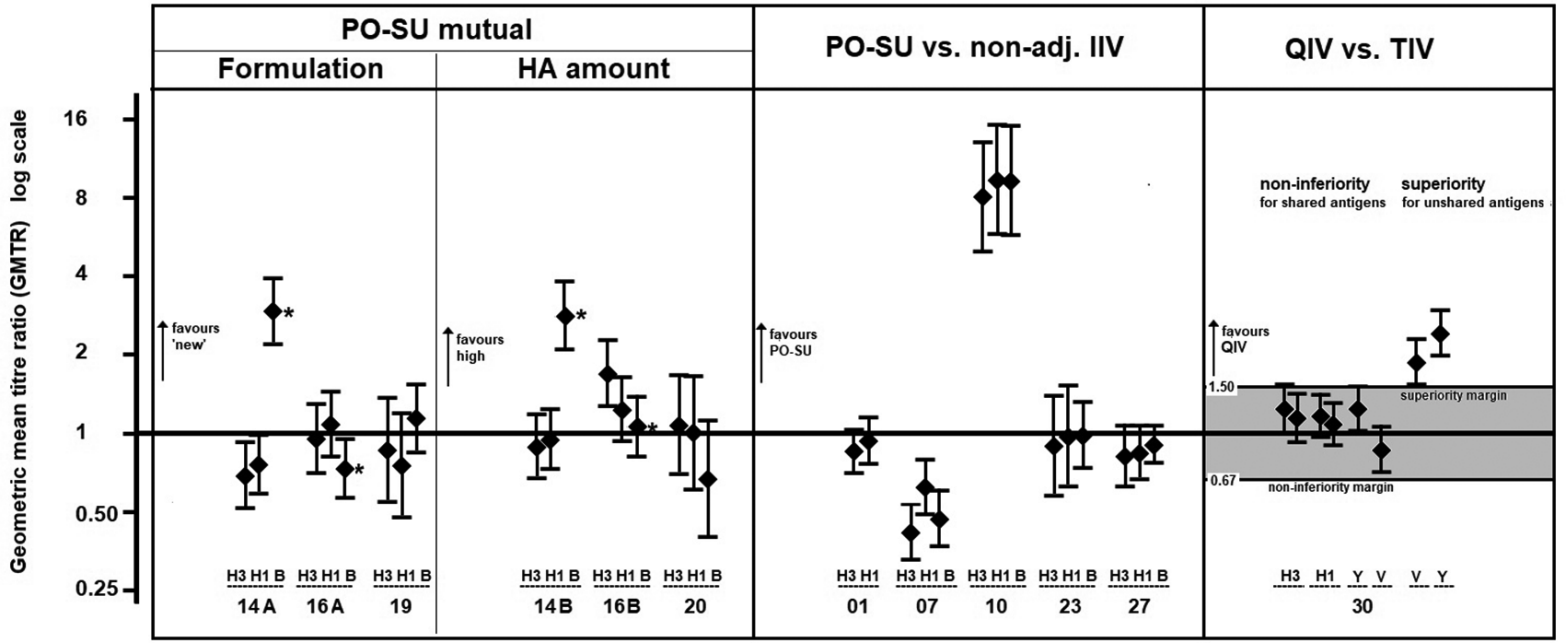
Randomised comparison trials (AZB-SU versus three comparator classes). Geometric mean titre ratios. Legend: AZB-SU mutual: T14A: AZB-SU _2008_ vs. AZB-SU _1996_; T16A: AZB-SU _TC_ vs. AZB-SU _1996_; T19: AZB-SU _TC_ vs. PO- SU _2008_; T14B: AZB-SU _2008_ 10 μg HA vs. AZB-SU _1996_ 5 μg; T16B: AZB-SU _TC_ 10 μg HA vs. AZB-SU _1996_; T20: AZB-SU _2008_ 10 μg HA vs AZB-SU _2008_. AZB-SU vs. non-adjuvanted inactivated influenza vaccine (IIV): T01, T07, T10: AZB-SU1 _996_; T23, T27: AZB-SU _2008_. QIV vs. TIV: QIV, quadrivalent influenza vaccine; TIV: trivalent influenza vaccine; Y, B/Yamagata; V: B/Victoria. Non-inferiority is demonstrated if the lower limit of the 95% confidence interval around the GMTR value (QIV versus TIV) is larger than the pre-defined non-inferiority margin of 0.67. Superiority is demonstrated if the lower limit of the 95% CI around the GMTR is larger than the pre-defined superiority margin of 1.5. * atypical influenza B component in 2008.

The immunogenicity analysis included data from 3311 participants and 9408 pre- and post-vaccination GMT pairs gathered from 28 intervention arms and 80 antibody sub-arms from 15 trials. A non-inferiority analysis comparing post-vaccination GMT values of AZB-SU and non-adjuvant comparator vaccines was performed using data from five trials ( Figure 4, middle panel). In three trials (Trial 01, Trial 23 and Trial 27), the 95% CIs of GMTR (AZB-SU: comparator) included 1, which indicated that the GMT values were not significantly different between treatment types. In Trial 10, the GMTR of all three viral strains had 95% CIs much higher than 1.0; AZB-SU vaccine GMT values were 8- to 9-fold higher than that of the comparator vaccine (AZB-SU: 239–448; comparator: 30–48). In Trial 07, the only trial performed in elderly participants (>60 years), 95% CIs were lower than 1.0 for all three strains. Comparator GMT values were 1 to 2-fold higher than those of AZB-SU ( Table 2). This result was explored further by evaluating whether the difference in antibody titre was associated with lower antibody-predicted clinical protection for AZB-SU vaccines. The post-PR _ab_ values ranged from 81.0% to 94.7% in AZB-SU treatment arms and from 87.5% to 97.8% in comparator arms. The respective ratios were close to 1.0, suggesting that protection was similar between AZB-SU and non-adjuvant comparator.

**Table 2.** Antibody and clinical protection levels for Trial 07, performed in the elderly.

(Sub)type	Intervention arm	Post-GMT	Post-protection rate (%)	Post-GMT ratio	Post-protection rate ratio
				(95% CI)	(95% CI)
A-H3N2	AZB-SU	148.4	89.9	0.42	0.94
	non-adjuvanted IIV	354.6	96.0	(0.33 to 0.53)	(0.88 to 1.00)
A-H1N1	AZB-SU	74.1	81.0	0.62	0.93
	non-adjuvanted IIV	118.7	87.5	(0.49 to 0.80)	(0.84 to 1.02)
B	AZB-SU	277.3	94.7	0.47	0.97
	non-adjuvanted IIV	587.2	97.8	(0.37 to 0.60)	(0.92 to 1.02)

CI = confidence interval; GMT = geometric mean titre; AZB-SU = polymer-adjuvanted subunit (vaccine).

A non-inferiority trial in adults aged 18–60 years compared the quadrivalent AZB-SU formulation with two trivalent non-adjuvant comparators (Trial 30), of which one contained B/Yamagata and the other contained B/Victoria. Non-inferiority of quadrivalent AZB-SU to trivalent AZB-SU was demonstrated for all three common strains ( Figure 4, right panel), and superiority of quadrivalent AZB-SU to trivalent AZB-SU for the B strain not included in the trivalent formulation.

The former re-licensing criteria of the CPMP needed to be evaluated in groups of 50 adult participants or more. ^20^ This requirement was met in 29 trial sub-arms from seven trials. Seroconversion rates, seroprotection rates and mean geometric increase after AZB-SU vaccination were higher than the CPMP thresholds set for adults aged 18–60 years and elderly adults in the majority of sub-arms; all 29 arms met at least one of these criteria ( *Extended data*, Figure H).

## Discussion

The current work analysing the available clinical evidence supports the hypothesis that across all age groups, the inclusion of azoximer bromide as an adjuvant to influenza subunit vaccine does not cause any increase in reported local or systemic reactions following vaccination. This conclusion particularly holds in elderly and vulnerable populations, the main target groups for yearly influenza vaccinations. Similarly, we noted that the antigen-sparing approach of including AZB and reducing total antigen (5 μg vs 15 μg HA per strain), resulted in similar antibody responses to non-adjuvanted vaccines in non-elderly patients; however, more data is necessary to make this conclusion for older populations.

Review of the available safety data suggests that AZB-SU vaccines are associated with fewer local reactions compared with vaccines that contained higher amounts of HA antigen; the incidence of systemic reactions, however, appears to be similar for AS-SU and other vaccines. This finding is congruent with observations made in large clinical trials, which showed an overall higher average rate of local reactions with higher HA per dose but little or no difference in the rate of systemic reactions. ^31^^,^^32^ The similarity in the systemic reaction rates between AZB-SU, placebo and comparator vaccines, suggests that the reporting of such systemic reactions was most likely to have been attributable to the act of intramuscular injection, or reflects little more than the everyday incidence of trivial symptoms that people experience; indeed, treatment arms in two trials (Trial 02 and Trial 08) had elevated R _sr_ values even though participants received no treatment. A symptomatic complex of systemic reactions following vaccination during pregnancy has been described, which was most often associated with psychological state and anxiety of the development of AEs in response to vaccination. ^33^

The intrinsic reactogenicity of AZB could not be determined directly, in the absence of randomised clinical trials comparing SU vaccines with identical amounts of HA, but with or without AZB. However, meta-regression analysis of the available data detected no difference in reaction rates after adjustment for mean age and the amount of HA administered, and predicted that AZB was not associated with intrinsic reactogenicity.

No SAEs of an allergic or neurological nature were reported in any of the trials selected for review, covering a total population of 11,736 participants. While this suggests a low risk of SAEs, a formal conclusion cannot be drawn as it exceeds the power of clinical trials to detect very rare events. However, favourable safety data come from a previous mass vaccination trial with AZB-SU that reviewed vaccination in nearly 420,000 paediatric participants and reported no more than 33 allergic SAEs (incidence: 0.008%; one event per 12,700 participants vaccinated) and no SAEs that were neurological in nature. ^34^

The immunogenicity data collected on more than 3000 participants across 15 studies generally supported the antigen-sparing effect of AZB, maintaining efficacy although the amount of HA in AZB-SU was only a third of the standard dose in comparable non-adjuvanted influenza vaccines. It was clear that AZB-SU vaccines induced antibody production in both children and adults up to 60 years at levels similar to those noted with comparator vaccines. This observation was seen for all comparisons except for one study in favour of AZB-SU (Trial 10; see Figure 4, middle panel) which remains unexplained but may result from a data artefact. The data from older adults (>60 years) were less robust, based on only three sub-arms. Analysis on post-PR _ab_ showed that clinical protection was not compromised in the AZB-SU vaccinees. Seroprotection and seroconversion data in AZB-SU treatment arms revealed that AZB-SU would have met CPMP criteria for annual re-licensing of vaccines in the European Union in both adults aged 18–60 years and adults >60 years.

A possible limitation of this study is its partial reliance on reports provided by the manufacturer. The sponsor’s clinical study reports had not undergone peer review, although the data from many of them were published in peer-reviewed journals. However, it is expected that these reports were prepared in line with good clinical practice, with a view to submission to regulatory agencies, and were therefore conducted robustly, countering the potential for any selection or interpretation bias. Review of the funnel plot data for reactogenicity variables showed no evidence of bias. In addition, during preparation of this article, two new studies assessed the safety and immunogenicity of the quadrivalent vaccine. Their findings are in line with those reported here, and they also provide supportive information on the practical use of quadrivalent AZB-SU vaccines. ^35^^,^^36^

Another possible limitation is the effect of pre-immunity and immune system status of the participants on their immune response to the vaccine and adjuvant. Natural antigenic drift results in different strains of influenza virus being prevalent annually. This results in individuals exhibiting a variable pre-immune status and response to vaccination that is dependent on the influenza strains they have previously encountered. The impact of this on vaccine and adjuvant effectiveness in this study is unknown. A study investigating MF59-adjuvanted influenza vaccine, found the effectiveness of adjuvant to diminish with the age of participants, with the greatest effect in children with minimal pre-immunity/prior exposure to flu. ^37^^,^^38^ Similarly, the potential presence or absence of chronic diseases on the health of participants and their response to the vaccine must also be considered. ^39^ The limited number of trials and vaccines investigated in this study was insufficient to form a conclusion regarding the role of either pre-immunity or health status on the participants’ response to the vaccines or adjuvants used. However, both present an avenue for future investigation.

In conclusion, the favourable safety profile and immunogenicity of AZB-SU vaccines, along with the reduced amount of antigen per dose and sparing effect of AZB, make AZB-SU vaccines good candidates for use not only during a pandemic or limited national capacity of vaccine production, but in general for seasonal influenza vaccination. Future research will be directed towards evaluating whether AZB also shows an antigen-sparing effect in elderly patients undergoing vaccination.

## Data availability

### Underlying data

Zenodo: Grippol Supplementary Files, https://doi.org/10.5281/zenodo.6221942. ^40^

This project contains the following underlying data:

- Grippol_Paper_Supplementary_Material.docx (Figure B; list of eligible publications)

### Extended data

Zenodo: Grippol Supplementary Files, https://doi.org/10.5281/zenodo.6221942. ^40^

This project contains the following extended data:

- Grippol_Paper_Supplementary_Material.docx
- README_file.txt.docx

### Reporting guidelines

Zenodo: PRISMA checklist for “Analysis of the safety and immunogenicity profile of an azoximer bromide polymer-adjuvanted subunit influenza vaccine”, https://doi.org/10.5281/zenodo.6221942. ^40^

Data are available under the terms of the Creative Commons Attribution 4.0 International license (CC-BY 4.0).
