# Supplementary material for: Analysis of the safety and immunogenicity profile of an azoximer bromide polymer-adjuvanted subunit influenza vaccine
Source: F1000Res. 2022 Nov 3;11:259. Originally published 2022 Mar 2. [Version 2] doi: 10.12688/f1000research.75869.2 (PMC9493399; doi:10.12688/f1000research.75869.2)
Supplement: Peer review history

# Peer review: Reviewer response for version 1

**DOI:** 10.5256/f1000research.79798.r149675

**Published:** 2022-09-21

**Authors:** Lyazzat Yeraliyeva

**Affiliations:** 1 Asfendiyarov National Medical University, Almaty, Kazakhstan

The relevance of the research topic is beyond doubt. Influenza is a serious disease causing various complications and deaths in infants, elderly people, and patients with chronic diseases. Vaccination is still the most effective method of influenza prevention.

 Today, there are 5 generations of influenza vaccines, and the authors carried out a systematic review and analysis of 30 clinical trials of various study designs, including 11,736 participants aged 6 months to over 60 years; the study was aimed at the reactogenicity, safety, and immunogenicity comparison of an inactivated adjuvanted influenza vaccine (AZB-SU vaccines) containing low antigen 5 µg HA per strain (with addition of azoximer bromide 500 µg) and non-adjuvanted vaccines containing antigen 15 µg HA.

 1. Abstract

 It is advisable to indicate in the abstract the meta-analysis period

 2. Introduction

 Provide a detailed information on the azoximer bromide adjuvant (Polyoxidonium), specifically its pharmacological properties.

 3. Methods

 In the "Definitions and outcomes" and "Statistical analyses" subsections, authors indicate the treatment outcomes, but the vaccination outcomes are required. To be clarified.

 4. Results

 It is necessary to clarify the period of meta-analysis carried out by the authors, since the period from 1993 to 2016 is indicated in the "Characteristics of studies" section, and 1998-2018 in the "Methods" section.

 5. Discussion

 It is necessary to discuss whether the presence of chronic diseases affected immunogenicity depending on the vaccine type used with different antigens and adjuvants content. Besides, it is necessary to discuss the immunogenicity issues

 Reviewing this article, I would like to note that the systematic review is of undoubted scientific and practical importance and will be of interest both among a wide range of medical workers, immunologists, epidemiologists, and among public health professionals.

Are the rationale for, and objectives of, the Systematic Review clearly stated?

Yes

Is the statistical analysis and its interpretation appropriate?

Yes

Are sufficient details of the methods and analysis provided to allow replication by others?

Yes

Are the conclusions drawn adequately supported by the results presented in the review?

Yes

Reviewer Expertise:

infection diseases, vaccination, epidemiology

I confirm that I have read this submission and believe that I have an appropriate level of expertise to confirm that it is of an acceptable scientific standard.

# Response

**Published:** 2022-10-31

**Authors:** Tim Hardman

**Affiliations:** Niche Science and Technology, UK

**1) Abstract**

**Comment:**

*It is advisable to indicate in the abstract the meta-analysis period*

**Response:**

 The meta-analysis period has been included in the draft of the manuscript, as recommended.

**2) Introduction**

**Comment 2:**

*Provide a detailed information on the azoximer bromide adjuvant (Polyoxidonium), specifically its pharmacological properties.*

**Response:**

 Thank you for your comment. A lot of the pharmacological information about azoximer bromide, including mechanism(s) of action and target(s) are still under investigation. However, the authorship believes all the relevant information has been included in the revised manuscript.

**3) Materials and methods**

**Comment:**

*In the "Definitions and outcomes" and "Statistical analyses" subsections, authors indicate the treatment outcomes, but the vaccination outcomes are required. To be clarified.*

**Response:**

 The vaccination outcomes are the treatment outcomes described. In this context ‘treatment outcome’ was used in a statistical sense, as opposed to the strict medical definition of the term, as this includes both the placebo and no-intervention arms.

**4) Results**

**Comment:**

*It is necessary to clarify the period of meta-analysis carried out by the authors, since the period from 1993 to 2016 is indicated in the "Characteristics of studies" section, and 1998-2018 in the "Methods" section.*

**Response:**

 The period of meta-analysis has been clarified in both the abstract and the results section.

**5) Discussion**

**Comment:**

*It is necessary to discuss whether the presence of chronic diseases affected immunogenicity depending on the vaccine type used with different antigens and adjuvants content. Besides, it is necessary to discuss the immunogenicity issues*

**Response:**

 This is another great, relevant point to raise and has also been added to the potential limitations of this study. The presence of chronic diseases and their impact on immunogenicity in the context of different antigens and adjuvants, are areas the authorship have involvement in, but again, the level of data available in this study was insufficient to investigate it here.

# Peer review: Reviewer response for version 1

**DOI:** 10.5256/f1000research.79798.r123089

**Published:** 2022-03-15

**Authors:** Marek Petra

**Affiliations:** 1 Department of Epidemiology and Biostatistics, Charles University Third Faculty of Medicine, Prague, Czech Republic

1) **Abstract**

 The main results were obtained by quantitative meta-analysis. It would be appropriate to state it also in the abstract.

 2) **Introduction**

 It will be suitable to better describe azoximer bromide, i.e. if it is used in some medical products, if it is a pharmacopoeial substance, etc.

 3) **Materials and methods**

 A meta-analysis was conducted, therefore the MOOSE assessment (Meta-analysis of Observational Studies in Epidemiology) is missing.

 In addition, the authors omitted the bias risk assessment according to the Grading of Recommendations Assessment, Development, and Evaluation (GRADE) guidelines. It is not clear if the study outcome can be burdened by this risk of bias.

 The fixed-effects method is applied in the case of studies' homogeneity. There is no explanation of the I-V method, i.e. the selection criteria. Furthermore, the random-effects method helps to evaluate the literary bias. Therefore, both results are required.

 4) **Results**

 Page 13, line 268: "The respective ratios included 1.0...", this should read: "The confidence interval of appropriate ratios included…".

 5) **Discussion**

 Influenza vaccine is a specific vaccine depending on seasonal influenza strains changing every year.

 Therefore, it should be discussed whether this specific feature of influenza seasons had an effect on the immunogenicity and, possibly, the safety of the adjuvanted influenza vaccine being evaluated.

Are the rationale for, and objectives of, the Systematic Review clearly stated?

Yes

Is the statistical analysis and its interpretation appropriate?

Yes

Are sufficient details of the methods and analysis provided to allow replication by others?

Partly

Are the conclusions drawn adequately supported by the results presented in the review?

Partly

Reviewer Expertise:

NA

I confirm that I have read this submission and believe that I have an appropriate level of expertise to confirm that it is of an acceptable scientific standard.

# Response

**Published:** 2022-10-31

**Authors:** Tim Hardman

**Affiliations:** Niche Science and Technology, UK

**1) Abstract**

**Comment:**

*The main results were obtained by quantitative meta-analysis. It would be appropriate to state it also in the abstract.*

**Response:**

 The initial wording of the abstract was unintentionally misleading and placed undue emphasis on the term ‘meta-analysis’. The main results of this study were obtained through systematic review and consisted of various point estimates from single trials/trial arms. The pooled rate differences discussed, and incorporated in Figure 3, are averages of the rate difference estimates from the involved trials, weighted by their inverse variance. This method was adopted over other techniques, such as unweighted average, and overall median, due to its superior accuracy. The results of this method are purely descriptive in nature, offering no particular conclusion beyond the expected result that the PO vaccine and comparators are similar in reactogenicity.

 The wording has been altered to more accurately portray the importance of anything related to meta-analysis.

**2) Introduction**

**Comment:**

*It will be suitable to better describe azoximer bromide, i.e. if it is used in some medical products, if it is a pharmacopoeial substance, etc.*

**Response:** Additional information has been added to the Introduction to briefly expand upon the usages of azoximer bromide.

**3) Materials and methods**

**Comment 1 *:*** *A meta-analysis was conducted, therefore the MOOSE assessment (Meta-analysis of Observational Studies in Epidemiology) is missing.*

**Response:** As mentioned in the earlier response, the term ‘meta-analysis’ was afforded undue importance. Originally the manuscript incorporated both a systemic review and meta-analysis, with the latter becoming less important and ultimately relegated into a hypothesis-creating tool as opposed to a central component of the study, as the significance of the data identified by the systematic review became more apparent.

 As such, the full MOOSE assessment was determined to be extraneous to the study in the context of the data from which the manuscript’s conclusions are drawn.

**Comment 2:** *In addition, the authors omitted the bias risk assessment according to the Grading of Recommendations Assessment, Development, and Evaluation (GRADE) guidelines. It is not clear if the study outcome can be burdened by this risk of bias.*

**Response:**

 As mentioned earlier, the importance of the meta-analysis in this manuscript was over-stated, with the results being generated by a systematic review. As such the bias risk assessment was not determined to be necessary for the finished manuscript.

**Comment 3:** *The fixed-effects method is applied in the case of studies' homogeneity. There is no explanation of the I-V method, i.e. the selection criteria. Furthermore, the random-effects method helps to evaluate the literary bias. Therefore, both results are required.*

**Response:**

 The I-V method, and random effects methods were both part of the meta-analysis, which, as mentioned, was relegated to a hypothesis-creating tool only.

**4) Results**

**Comment:**

*Page 13, line 268: "The respective ratios included 1.0...", this should read: "The confidence interval of appropriate ratios included…".*

**Response:**

 Thank you for pointing out this oversight, a more accurate phrasing of the sentence that is closer to its original intent, has been drafted.

**5) Discussion**

**Comment:**

*Influenza vaccine is a specific vaccine depending on seasonal influenza strains changing every year.*

*Therefore, it should be discussed whether this specific feature of influenza seasons had an effect on the immunogenicity and, possibly, the safety of the adjuvanted influenza vaccine being evaluated.*

**Response:**

 This is an excellent point to raise and has been added to the potential limitations of this study. Members of the authorship have conducted some investigations into the impact of these features of influenza in the context of a different adjuvant, but the level of data available in this study was insufficient to investigate it here.
